# Role of Adaptive and Innate Immunity in Type 2 Diabetes Mellitus

**DOI:** 10.1155/2018/7457269

**Published:** 2018-11-08

**Authors:** Tong Zhou, Zheng Hu, Shuo Yang, Lin Sun, Zhenxiang Yu, Guixia Wang

**Affiliations:** ^1^Department of Endocrinology and Metabolism, The First Hospital of Jilin University, Changchun, Jilin Province, China; ^2^Institute of Translational Medicine, The First Hospital of Jilin University, Changchun, Jilin Province, China; ^3^Department of Respiration, The First Hospital of Jilin University, Changchun, Jilin Province, China

## Abstract

After the recognition of the essential role of the immune system in the progression of type 2 diabetes mellitus, more studies are focused on the effects produced by the abnormal differentiation of components of the immune system. In patients suffering from obesity or T2DM, there were alterations in proliferation of T cells and macrophages, and impairment in function of NK cells and B cells, which represented abnormal innate and adaptive immunity. The abnormality of either innate immunity, adaptive immunity, or both was involved and interacted with each other during the progression of T2DM. Although previous studies have revealed the functional involvement of T cells in T2DM, and the regulation of metabolism by the innate or adaptive immune system during the pathogenesis of T2DM, there has been a lack of literature reviewing the relevant role of adaptive and innate immunity in the progression of T2DM. Here, we will review their relevant roles, aiming to provide new thought for the development of immunotherapy in T2DM.

## 1. Introduction

Type 2 diabetes mellitus (T2DM) is characterized by abnormally elevated levels of blood glucose due to impaired insulin secretion, glucose intolerance, and hyperglycemia. It is also considered as a major burden for healthcare systems worldwide [1]. Nowadays, the pathogenesis of T2DM is considered to be linked to both innate and adaptive immune factors that are recognized as important etiological components in the development of insulin resistance [2]. Epigenetic mechanisms controlling immune cell lineage determination, function, and migration are implicated in obesity and T2DM. Obesity is associated with low-grade inflammation and is responsible for the activation of immune system in patients suffering from T2DM [3]. Increased fat mass in obese patients can induce metabolic dysregulation. It has also been recognized that insulin resistance during obesity is closely related to adipose tissue inflammation [4]. The abnormal proliferation of factors of innate and adaptive immune system was observed during adipose tissue inflammation that may lead to the development of T2DM [3]. For example, impaired NK cell function was observed, particularly with respect to NKG2D expression, which was negatively correlated with HbA1c levels [5]. Moreover, polarization of macrophage to M1 was upregulated, and activation of CD4+ T lymphocytes expressed in visceral adipose tissue of obese mice was elevated [6–8]. Since previous studies have reviewed the functional involvement of T cells in progression of T2DM, or regulation of metabolism by the innate immune system or adaptive immune system during the pathogenesis of T2DM, literature reviewing the relevant role of adaptive and innate immunity in the progression of T2DM in recent years still remains scarce. Therefore, here, we will review the relevant research, aiming to provide new ideas for the subsequent research.

## 2. T Cells in T2DM

In recent years, it has been reported that T cells play key roles in the progression of T2DM. It indicated that T2DM was associated with overactivated T cells and the activation of the inflammatory pathways [8]. Differentiation of effector T cells is tightly related by the regulation process that influences the production of distinct sets of effector cytokines [9]. T cells are abnormally differentiated in T2DM patients.

### 2.1. CD4+ T Cells

CD4+ T cells play an important role in the pathology of obesity and insulin resistance. CD4+ effector T cells can be further subdivided into proinflammatory Th1 and Th17 and anti-inflammatory Th2 and Foxp3+ regulatory T (Treg) cell subtypes based on their functionality and the type of cytokine produced [10]. It was reported that the percentage of CD45+ leukocytes increased significantly in obese patients with or without T2DM [11]. The balance between Th2 or Treg and effector T cell subsets such as Th1 or Th17 cells is important for immune homeostasis and the immune response. A large amount of evidence has showed that there was an imbalance in the differentiation in CD4+ T cells in obese T2DM patients.

#### 2.1.1. T Helper (Th) Cells

Th1 cells are differentiated CD4+ T cells characterized by the production of proinflammatory interferon-*γ* (IFN-*γ*) [12]. Th2 cells are differentiated from activated CD4+ T cells, which drive the production of key Th2 cell lineage-defining cytokines such as interleukin- (IL-) 4, IL-5, and IL-13 [13]. When CD4+ T cells are encountered by IL-6 and transforming growth factor-*β* (TGF-*β*), they tend to differentiate to Th17 cells, which play a pathogenic role in various inflammatory disorders [14].

CD4+ T cells tend to polarize to proinflammatory Th1 cells and Th17 cells in peripheral blood and adipose tissue in patients suffering from T2DM. In contrast, polarization of anti-inflammatory Th2 cells was decreased [15–20]. A recent study [21] showed that Th1/Th2 ratio and levels of cytokines (e.g., IL-4, IL-10, IL-13, and IFN-*γ*) were significantly elevated, whereas nuclear factor erythroid 2-related factor 2 (Nrf2) and its downstream targets, which act with antioxidant, detoxification, and maintenance of cellular redox homeostasis and glutathione homeostasis and influence mitochondrial biogenesis, were decreased in T2DM patients. The circulatory levels of Nrf2 showed a positive correlation with the levels of Th2 cytokines and negative correlation with the levels of Th1 cytokines [21]. In addition, it showed that CD4+ and CD8+ T cells were infiltrated in both visceral adipose tissue (VAT) and subcutaneous adipose tissue (SAT), with proinflammatory Th1 and Th17 cells significantly more frequent in VAT as compared with SAT. The frequency of Th1 cells in SAT and VAT correlated directly with plasma hsCRP concentrations, while the frequency of Th2 correlated inversely with plasma hsCRP concentrations. Furthermore, Th1 cell frequency in SAT also correlated with plasma IL-6 levels [15, 22].

The secretion of cytokines (tumor necrosis factor- (TNF-) *α*, IFN-*γ*, and IL-17) and T lymphocytes was drastically upregulated during the pathogenesis of obesity-induced insulin resistance and development of T2DM [23–25]. With respect to the relationship between frequency of lymphocytes and activation of disease, it was found that the frequency of IFN-*γ*-producing CD3+ T cells was positively correlated with body mass index (BMI) [26]. Previous studies have reported that the frequency of cytokines can be influenced by other types of lymphocytes. B cells support Th17 inflammation in T2DM patients but not in the normal people, whereas monocytes support Th17 inflammation regardless of whether an individual is suffering from T2DM [27]. In addition, the cytokines produced by Th1, IL-2, and TNF-*β*; the cytokines produced by Th17, IL-17F, and IL-17A; and the cytokines produced by both Th1 and Th17 cells are important for explaining the HbA1c variance [28]. During the progression of T2DM, levels of mRNA and protein of IL-2 and TNF-*α* in diabetic retinopathy (DR) patients were dramatically higher compared with those of the healthy control group [29]. Moreover, the DR group showed higher IL-2 and TNF-α levels, however lower IL-4 and IL-10 levels than T2DM group without DR [26, 28]. Zhang et al. [30] discovered that levels of vascular endothelial growth factor (VEGF), which is related to the progression of diabetic microvascular disease, were positively correlated with Th1 percentage and the ratio of Th1/Th2. They also found that Th1/Th2 ratio was an independent predictor of VEGF levels in T2DM patients [30]. Angiotensin II increases the activities of T cells and the expression of IFN-*γ* and IL-17 significantly during the development of carotid atherosclerosis in patients suffering from T2DM [31].

#### 2.1.2. Treg Cells

Treg cells express CD4, CD25, and forkhead family transcription factor Foxp3. They represent a small subset of T lymphocytes constituting only 5–20% of the CD4+ compartment, suppressing effector T cell responses, limiting inflammation, and preventing autoimmunity [14, 32, 33]. CD4+ T cells tend to polarize to Treg cells *in vitro* when treated with TGF-*β*, which appears to be dependent upon fatty acid oxidation and cholesterol metabolism rather than glycolysis [34, 35]. Treg cells suppress the levels of effector T cells by various pathways [36], including the inhibition T cell receptor- (TCR-) induced proliferation and IL-2 transcription of conventional T cells (Tcons) [37], the release of anti-inflammatory cytokines like IL-10 and TGF-*β* [38], the expression of the coinhibitory molecule cytotoxic T lymphocyte antigen-4 (CTLA-4) [39], the expression of the hallmark transcription factor of suppressed CD4+ T cells [40], and the ability to migrate by activation of glycolysis [41].

The balance between Treg and Th1 or Th17 cells is important for immune response in T2DM patients. Treg cells suppress the activities of Th1, Th2, and Th17 cells to improve insulin resistance. It has been reported that the percentage of Treg cells was decreased in peripheral blood of T2DM patients, especially in newly diagnosed patients, which culminates in the progress of inflammation and insulin resistance [42, 43]. It was reported that both Treg/Th17 ratio and Treg/Th1 ratio decreased in patients suffering from T2DM [44]. High levels of insulin in a model of diet-induced obesity impaired the ability of Tregs to suppress inflammatory responses via the AKT/mTOR signaling pathway, which showed lower expression of IL-10 and poor ability to suppress the production of TNF-*α* [45]. In obese patients also suffering from T2DM, an even lower percentage of Treg cells was revealed [46].

CD39+ Treg cells are responsible for suppressing Th17 cells and are believed to be reduced in number in obese T2DM patients [47]. It was confirmed that CD39 was related to the stability and the suppressive function of Treg cells [19]. It also indicated that, in overweight and obese T2DM patients, the levels of CD4+ IL-17+ cells showed a positive correlation with blood glucose and HbA1c expression levels. CD39^hi^ Treg cells express higher CTLA-4 and produce more IL-10 than CD39^low^ Tregs [19, 45, 47].

### 2.2. CD8+ T Cells

CD8+ T cells are essential for the adaptive immune response against infections by secreting cytokines, such as IFN-*γ* and TNF-*α*. It is well established that CD8+ T cells can also synthesize and express the proinflammatory cytokine IL-17, which is present in inflammatory tissues in various human inflammatory diseases [48]. Previous literature has shown that the accumulation of these cells induces inflammation and insulin resistance [49]. Patients with T2DM or mice with high-fat diet (HFD) seem to have higher percentage of CD8+ cytokine T cells [49–53]; however, the proportion of CD8+ T cells decreased after 120 min of glucose loading [54]. Pathogenic CD8+ T cell subsets, which control hepatic insulin sensitivity and gluconeogenesis, accumulate in the liver of diet-induced obese mice [55]. The accumulation was supported by type I interferon (IFN-I) responses [55]. It has been reported that the concentration of IFN-*γ* positively correlated with BMI of T2DM patients. In obese individuals, the levels of IFN-*γ* produced by CD8+ T cells increased when compared with lean individuals, which might modulate the insulin resistance [56]. Diet-induced obesity did not affect the maintenance of preexisting memory CD8+ T cells, including acquisition of a long-term memory phenotype and function [57].

### 2.3. NKT Cells

NKT cells refer to a large fraction of NK marker+ T cells (“NKT”) which recognize the major histocompatibility complex (MHC) class I-like CD1d protein expressed on the surface of antigen-presenting cells and use an identical “invariant” TCR*α* chain [58, 59]. Three subgroups of NKT cells can be distinguished according to their antigen specificity and TCR: invariant NKT (iNKT), type II NKT, and NKT-like lymphocytes [60]. iNKT cells are present in large numbers in adipose tissue. Upon activation, iNKT cells can secrete high levels of cytokines, including IL-4 and IFN-*γ*. But as adipose tissue expands during obesity, the number of iNKT cells decreased, correlating with proinflammatory macrophage infiltration. Mice lacking iNKT cells, when fed on HFD, showed higher weight, larger fat pads, elevated fasting blood glucose (FBG), impaired glucose tolerance test (GTT), and increased insulin resistance compared to wild-type (WT) mice. After weight loss, the abundance of iNKT cells was restored [61], followed by improvement in glucose tolerance and insulin sensitivity. The activation of NKT cells, influenced by M1 macrophages, promoted Th1 responses and inhibited M2 polarization. The expression of CD1d in M2 macrophages could activate NKT cell-mediated immune responses and disrupts the immune balance [62]. The depletion of NKT cells enhances the presence of M1 macrophages in visceral AT and increases insulin resistance and glucose intolerance [63]. It was reported that there was decreased frequency of iNKT cells in VAT and peripheral blood of obese patients [64]. Despite the several studies analyzing the frequency of iNKT cells in T2DM patients, no specific conclusion has been drawn yet [50, 65]. While recent studies identified that the gut microbiota may play a crucial role in iNKT cell development, it can be speculated that T2D alterations of the gut microbiota, and possibly even AT-associated microbiota, affect iNKT cell homeostasis in the gut and AT [66].

## 3. B Cells in T2DM

B cells have been shown to play a central role in the development of insulin resistance [67], through the production of IgG antibodies and the activation of T cells and macrophages [68]. There were no significant differences between the percentage of B cells in peripheral blood mononuclear cells (PBMCs) of T2DM or obese patients and healthy patients [11]. However, van Beek et al. showed that the expression of activation marker CD38 of B cells was significantly higher on circulating B cells in the obese patients with normal glucose tolerance as compared to obese patients suffering from T2DM [69]. Besides, DeFuria et al. argued that HFD induced a significant increase in the proliferation of B cells in VAT [70]. In patients with obesity or T2DM, DNA methylation, which is likely to be an important mechanism contributing to the interindividual variation in function of immune cells, was proved to stimulate the proliferation of B cells [71]. As we had discussed before, B cells could affect the proliferation of Th17 and the production of proinflammatory cytokines in T2DM patients. It was found that on depletion of CD19+ cells, Th17 proliferation was decreased in T2DM patients but not in individuals without T2DM [27]. Not only obese T2DM patients but also nonobese T2DM patients exhibited significantly high levels of fecal IgG, which is produced by B cells, compared to control individuals with similar BMI [70, 72]. The mice that failed to produce mature B cells showed lower fasting glucose levels and improved glucose tolerance when fed with HFD for 8 weeks compared to HFD-fed WT mice [70]. These results indicated that B cells participated in the process of promoting insulin resistance and glucose intolerance by activating Th1 and Th17 cells and releasing pathogenic antibodies.

## 4. NK Cells in T2DM

NK cells could recognize and deal with both tumors and viral or bacterial infections, all of which are prevalent in T2D. NK cell dysfunction was considered to lead to an increased risk of several infections and several cancer types [73]. The activity of NK cells is regulated by activating receptors, including NKp30, NKp44, NKp46, NKG2C, and NKG2D, all of which bind ligands present at the surface of tumor cells or infected cells [74]. NK cells could be divided into two main subtypes, one subgroup which mainly plays a role in cytotoxicity by expressing CD56dim CD16bright and another subgroup that mainly secretes cytokines (e.g., TNF*α*, IFN-*γ*, IL-8, and IL-10) and expresses CD56bright CD16dim/negative [75]. The number of total NK cells in obese or T2DM patients has exhibited discrepancies among different studies. Some studies have found that obese or T2DM patients exhibited higher number and/or activation of NK cells in the circulation or adipose tissue compared to control subjects [76–78], while a few other studies observed corresponding decreases or no changes at all [79]. Although it is generally recognized that the number of highly cytotoxic CD56dim NK cells decreased in obese individuals, the number of low cytotoxic CD56bright NK cells increased, along with the cytokines, including NKG2D and IFN-*γ*, produced by NK cells [80]. Berrou et al. has demonstrated that T2DM patients have a decreased frequency of NKp46- and NKG2D-positive NK cells and defects in NK cell function, which was evident from reduced degranulation [5]. Patients with uncontrolled diabetes had the lowest levels of NKG2D expression, and there is a significant inverse correlation between NKG2D-expressing NK cells of diabetic patients and their HbA1c levels. In VAT of HFD mice, NK cells skew macrophage differentiation into a proinflammatory M1 phenotype which further promotes inflammation and the development of obesity-induced insulin resistance [81].

## 5. Myeloid Cells in T2DM

Innate immunity also includes macrophages, monocytes, neutrophils, eosinophils, and basophils. Macrophages were emphasized to play a key role in the pathological progress of obesity or insulin resistance. Two subtypes of macrophages are present in the adipose tissue: a proinflammatory macrophage type, termed “M1” cultured in the presence of GM-CSF, and an anti-inflammatory macrophage type, termed “M2” in the presence of IL-4. M1-like macrophages showed proinflammatory effects by secreting high levels of proinflammatory markers and cytokines such as TNF, whereas M2-like macrophages exhibited increased secretion of anti-inflammatory cytokines such as IL-4 and IL-10 [82]. Macrophages infiltrated into expanding adipose tissue causing inflammation and linking obesity to insulin resistance. In obese humans, AT macrophages displayed profound proinflammatory (M1) polarization, under the influence of local environmental factors within the adipose tissue, and are thereby thought to be the major source of proinflammatory cytokines and chemokines [83, 84]. These proinflammatory macrophages also secrete chemokines to recruit the next wave of incoming monocytes. Loss of peroxisome proliferator-activated receptor-*γ* (PPAR*γ*) (induced by IL-4) expression in macrophages can also result in impaired insulin sensitivity in the liver and muscle [85]. Disruption of PPAR*γ* in myeloid cells impairs alternative macrophage activation (M2-like macrophage), thereby playing a key role in the development of diet-induced obesity, insulin resistance, and glucose intolerance.

Furthermore, macrophage activation is mediated by cells involved in adaptive immunity. Th1 and Th17 cells release IFN-*γ* and IL-17, which stimulate proinflammatory M1 macrophage differentiation, whereas Th2 cells and Treg cells promote anti-inflammatory M2 macrophage polarization via the production of IL-4, IL-10, or IL-13 [23, 86]. Macrophages in adipose tissue were considered as final effector cells, which regulate adipose tissue inflammation [81]. IL-6, which was reported to be increased in obese individuals, and promoted the development of insulin resistance and T2DM [87], could be secreted from macrophages and adipocytes in adipose tissue [88]. Kim et al. reported that expression of lamin A/C, which is a protein meshwork that surrounds and protects the nuclear content, was upregulated in adipose tissue in obese and type 2 diabetes patients. Moreover, lamin A/C expression is specifically upregulated in adipose tissue macrophages (ATMs), particularly in CD11c+ M1 ATMs, due to obesity, which could further promote expression of proinflammatory cytokines by enhancing activity of nuclear factor kappa-light-chain-enhancer of activated B cells (NK-*κ*B) [89].

Beside altered macrophages, monocytes also play a key role in the progression of obesity and insulin resistance in T2DM patients. Patients suffering from T2DM exhibited increased expression of monocyte activation markers (CD11b and CD36). Monocyte surface CD163 expression levels were significantly associated with insulin resistance in type 2 diabetes patients, which showed the pathophysiological role of monocyte CD163 in the development of insulin resistance [90]. Eosinophils and neutrophils also play important roles in the immune regulation of T2DM. T2DM patients exhibited significant decrease in the number of neutrophils and eosinophils [17, 91]. Mice with restored eosinophilia (overexpressing IL-5) demonstrated decreased adiposity and improved insulin sensitivity when fed a high-fat diet [91].

## 6. Immunotherapy in T2DM

Recently, there have been several studies focusing on immune effect of hypoglycemic drugs or the improvement of glucose and lipid metabolism using immune system modulators. Metformin, which was initially recommended as the drug for T2DM treatment [92], has also been found to downregulate the mRNA levels of NK-kB and IL-1*β*, thus increasing the insulin sensitivity [93]. Metformin could influence macrophages as well by inducing the expression of reactive oxygen species in macrophages, which led to a phenotypic shift in macrophages toward M2-like macrophages via a partially adenosine monophosphate-activated protein kinase- (AMPK-) independent manner [94].

Consistent with these results, oral anti-CD3 plus glucosylceramide (an NKT cell target antigen) treatment has been shown to induce the production of IL-10 and TGF-*β*, which were associated with improved levels of glucose while fasting, visceral adipose tissue inflammation, liver enzymes, and hepatic steatosis in ob/ob mice [95]. Similar effects were observed by the induction of TGF-*β*-dependent CD4+ latency-associated peptide (LAP)-positive Tregs, which decreased the number of CD11b+ F4/80+ macrophages and TNF-*α* in adipose tissue of leptin-deficient ob/ob mice [96]. Activation of Nrf2 which was reported to be downregulated in PBMCs of T2DM patients, along with skew Th1 and Th2 dominance, could restore cytokine stress and the impaired insulin secretion in pancreatic *β*-cells [97]. In addition, there have been studies on transgenic mice with respect to the proliferation and function of components of immune system. OX40-KO mice exhibited significantly less body weight and lower fasting glucose levels than WT mice, without obvious adipose tissue inflammation. OX40 deficiency suppresses CD41+ T cell activation and prevents macrophage infiltration in the adipose tissues of obese mice [97]. Li et al. introduced a recombinant adenovirus carrying the protein tyrosine phosphatase nonreceptor type 2 (*PTPN2*) gene into epididymal white adipose tissue (EWAT) of ApoE−/− mice. The adenovirus reversed the high Th1/Treg and Th17/Treg ratios, macrophage infiltration, the ratio of M1/M2 macrophages, and the expression of proinflammatory cytokines in EWAT of diabetic mice [98].

## 7. Conclusion

**Figure 1:**
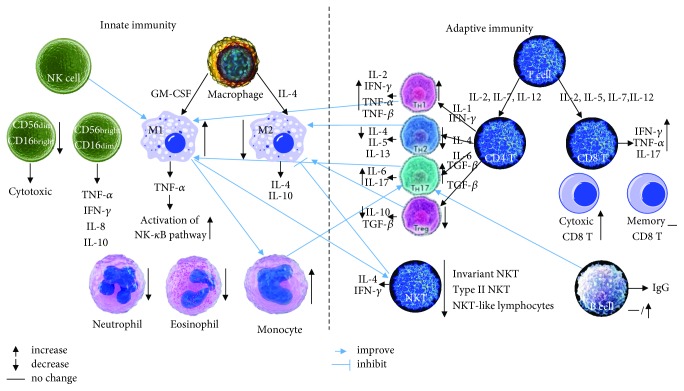
Role of innate immunity and adaptive immunity in the condition of T2DM, obesity, or adipose tissue of HFD mice.

Since there were many similarities in the pathological progress of obesity and T2DM, which are tightly linked, altered proliferation, function, or infiltration of components of adaptive immunity and innate immunity is critical in the progression of T2DM. Obesity and T2DM share similar T cell compartment alterations that may contribute in the metabolic disturbances associated with them. These alterations include increased number of CD45+ T cell, leukocyte shift toward a proinflammatory phenotype, and a reduction in the number of suppressive regulatory T cells and protective NK cells. A higher susceptibility to infections has been observed in patients suffering from obesity and T2D, which indicates a T cell homeostatic dysregulation, NK cell dysfunction, and abnormal polarization of macrophages (Figure 1). Metabolic alterations in patients suffering from obesity and T2DM may further affect differentiation, function, and survival of components of innate immunity and adaptive immunity. Adipokines, such as leptin, increase T cell proliferation and Th1/Th17 cytokine secretion and prevent apoptosis via the mTOR signaling pathway following antigen stimulation [99].

The cross-talk and dynamics of immune cells initiating and orchestrating AT inflammation and an impaired lipid metabolism at different AT depots in the obese individuals who do not have diabetes or are suffering from type 2 diabetes are still not completely understood. The signals and mechanisms involved in obesity- and/or T2D-mediated modulation of T cell functions remain poorly documented. The mechanisms explaining change in the composition of cytokines secreted by different types of immunocytes are required to be elucidated. There have not been many studies related relationships of immune system and diabetes. The development of a personalized immunotherapy by identifying the metabolic immune checkpoint is highly desirable.

Above all, there has been increasing number of studies linking the immune system to T2DM. It was considered whether T2DM was an autoimmune disease because of the importance of inflammation in the development of insulin resistance, obesity, and T2DM. It is necessary to understand the role of innate immunity and adaptive immunity in obesity and diabetes, which could reveal novel immunotherapeutic approaches to modulate metabolic inflammation and insulin resistance.

## Abbreviations

ATMs: Adipose tissue macrophages
AMPK: Adenosine monophosphate-activated protein kinase
BMI: Body mass index
CTLA-4: Cytotoxic T lymphocyte antigen-4
DR: Diabetic retinopathy
EWAT: Epididymal white adipose tissue
FBG: Fasting blood glucose
GTT: Impaired glucose tolerance test
HFD: High-fat diet
IFN-*γ*: Interferon-*γ*
IFN-I: Type I interferon
IL-4: Interleukin-4
IL-5: Interleukin-5
IL-6: Interleukin-6
IL-10: Interleukin-10
IL-13: Interleukin-13
IL-17: Interleukin-17
iNKT: Invariant NKT
MHC: Major histocompatibility complex
NK-kB: Nuclear factor kappa-light-chain-enhancer of activated B cells
Nrf2: Nuclear factor erythroid 2-related factor 2
PBMC: Peripheral blood mononuclear cell
PPAR*γ*: Peroxisome proliferator-activated receptor-*γ*
PTPN2: Protein tyrosine phosphatase nonreceptor type 2
SAT: Subcutaneous adipose tissue
T2DM: Type 2 diabetes mellitus
Tcons: Conventional T cells
TCR: T cell receptor
TGF-*β*: Transforming growth factor-*β*
Th cell: T helper cell
TNF-*α*: Tumor necrosis factor-*α*
TNF-*β*: Tumor necrosis factor-*β*
Treg cell: Regulatory T cell
VAT: Visceral adipose tissue
VEGF: Vascular endothelial growth factor
WT: Wild type.
